# Local Implementation of Cancer Control Activities in Rural Appalachia, 2006

**Published:** 2008-12-15

**Authors:** Bruce Behringer, Karen Harrell Mabe, Kelly A. Dorgan, Sadie P. Hutson

**Affiliations:** Office of Rural and Community Health and Community Partnerships, East Tennessee State University; Community Cancer Research Review Work Group, Johnson City, Tennessee; East Tennessee State University, Johnson City, Tennessee; East Tennessee State University, Johnson City, Tennessee

## Abstract

Underserved communities with high cancer rates often are not involved in implementing state cancer control activities locally. An East Tennessee State University research team formed 2 Appalachian Community Cancer Research Review Work Groups, 1 in northeast Tennessee and 1 in southwest Virginia. During 4 sessions, the research team presented regional cancer data to the work groups. Work group participants explored research from a lay perspective and identified possible reasons for cancer disparities in central Appalachia. The fifth session was a community dissemination activity in which work group participants engaged in cancer education and action by presenting the research to their local communities in unique ways. During a sixth session, both work groups discussed these interventions and further attempted to answer the question, "What makes the experience of cancer unique in Appalachia?" This article describes the key steps of this community-based participatory research process.

## Background

Appalachia — the mountainous region of 13 states in the eastern United States — suffers from disproportionately high rates of cancer (1), but leaders of state cancer coalitions in the 13 states have been challenged to implement cancer control activities recommended by the National Comprehensive Cancer Control Program of the Centers for Disease Control and Prevention (CDC) (2). Residents who wish to implement cancer-related health promotion and cancer prevention activities in their communities may be unable to access or understand research findings or may find them confusing and conflicting. Our research team collaborated with informal leaders in these communities in 2006 by forming Community Cancer Research Review Work Groups. The work groups were a mechanism to present regional cancer data to community members, generate discussion about those data, and empower participants to return to their communities with education activities. Presenters used numbers (quantitative data) and narratives (qualitative data) to describe health disparities in Appalachia and to discuss the strong influence of communication, culture, and community on cancer (3). People in mountain communities historically have perceived that their communities have higher rates of cancer incidence and mortality than do other regions (4). Current research now partially supports this perception (5,6).

## Implementation

We organized 2 Community Cancer Research Review Work Groups, 1 in northeast Tennessee and 1 in southwest Virginia. Our years of involvement in cancer education and outreach allowed us to identify unofficial community leaders who were interested in cancer issues. Our goal was to follow the precepts of community-based participatory research (CBPR) (7) to bring findings from previous studies to the community for interpretation from a lay perspective. The work groups 1) reviewed findings and considered how those findings applied to their community, 2) recommended how to best present those findings in ways their community would accept and act on, and 3) identified what makes the cancer experience unique in Appalachia. The work group approach was submitted to and approved by the East Tennessee State University (ETSU) institutional review board. The program research design and approach were explained to participants, who gave informed consent.

### Step 1: Identify the right people for engagement

In an effort to understand the disparity in cancer rates, we recruited unofficial community leaders who were concerned about cancer to join the work groups. These community leaders helped us select work group participants with vital community connections and a personal interest in cancer. Credible cancer communication partners, especially during recruiting, are needed to conduct CBPR in Appalachian and other rural communities (8). We identified and recruited people who had personal experience with cancer (eg, survivors, caregivers) and other volunteers who were able to understand and discuss opinions about research (eg, educators, community advocates, church leaders). We intentionally did not select participants who were health professionals. All participants were willing to interpret and present research findings within their communities. The work groups were successful because the mix of community members represented a blend of diverse voices. Careful recruitment of the 24 participants (14 in Tennessee and 10 in Virginia) ensured diversity of professional backgrounds, educational levels, and demographic characteristics. Participants ranged in age from 30 to 65 years and included preachers, a funeral home director, current and retired school teachers, a librarian, a lawyer, housewives, and community activists. White, African American, and Hispanic community members participated, as did men and women of all ages. Work group moderators (K.A.D. and S.P.H.) reminded participants that they were not representing any particular demographic group, organization, or program. Instead, the moderators asked participants to focus on their understanding of cancer as a community problem and blend a variety of viewpoints from their communities.

### Step 2: Establish a climate for learning together

During the work group meetings, 1 common question participants raised was, "What makes us [Appalachians] unique?" In addressing this question, both researchers and participants examined qualitative and quantitative research and identified possible characteristics, such as personal behaviors, literacy levels, and health care access. The work group approach provided a shared time and place for participants and researchers to discuss these factors within a regional framework.

First, the research team encouraged a "co-learning" climate. The project title, Community Cancer Research Review Work Groups, indicated the importance of participants' roles. We designed each work group session to present cancer research in an understandable way and allowed sufficient time for participants to question and interpret the studies.

Second, the tone set during the work group sessions ensured that all voices were heard. Moderators created an environment where community members freely examined the research and offered interpretations. For example, participants explained that the belief that "Cancer is a death sentence" (voiced in earlier focus groups of the Rural Appalachian Cancer Demonstration Program) (9) was held largely on the basis of Appalachian experiences associated with late-stage cancer diagnoses common in rural communities. Participants challenged the idea that "Appalachian fatalism" was fading or perhaps was being replaced by fatalism over an inability to effectively access the health care system. These types of alternative explanations and interpretations are an example of empowerment evaluation (10), an approach that directly involves communities in improving researchers' findings and translating findings into effective programs.

Third, the research team recognized that community members often cannot commit to long-term action and, therefore, required only a short-term commitment from participants. This respect of participants' busy schedules may have encouraged high rates of attendance (20 hours, on average, over the course of 3 months, including the five 2-hour work group sessions). The Table outlines the content of the 6 work group sessions. Sessions 1 through 4 were 2 hours and were facilitated by ETSU faculty members (K.A.D. and S.P.H.). For session 5, participants used information from sessions 1 through 4 to design cancer education events or activities in their communities, which they then organized and implemented at times convenient to community members shortly following sessions 1 through 4 during May 2006. For session 6, the work groups met together to report their activities and to further discuss the research from an Appalachian community perspective.

### Step 3: Help participants develop new skills to be good consumers of cancer research

Community members knew that cancer dramatically affects their families and neighborhoods, but most were unaware of their actual "cancer numbers" (eg, incidence and mortality rates for their communities and national statistics on survivorship by type of cancer) (11). We presented cancer data in a variety of ways. Guest speakers began the first 4 sessions, presenting research findings during the first hour. During the second part of sessions 1 through 4, moderators (K.A.D. and S.P.H.) facilitated a discussion of work group member interpretations of the research. Participants learned new ways of questioning data, examining individual and collective community experiences, and generating group interpretations that began to create an Appalachian cultural lens for cancer.

In Session 1, a researcher (B.B.) presented color-coded maps from disparities studies and localized data tables, so participants could visually compare cancer rates in their region with state and national rates. Data were displayed by cancer type and by sex and age group of people diagnosed with cancer. Participants were able to examine objectively whether their intuition about cancer in their communities was accurate and to increase their understanding of Appalachian cancer disparities. Session 2 introduced a series of quantitative studies from the literature, the Appalachian Regional Commission, and the Rural Appalachian Cancer Demonstration Program qualitative studies (1,5). These findings reinforced participants' concerns about barriers to effective communication between patients and health care providers, such as talking about cancer with physicians, including physicians from foreign countries. Participants cited their own communication experiences during stressful events, such as receiving a cancer diagnosis. Participants said health professions schools and health systems should do a better job of teaching and using culturally appropriate communication.

Session 3 introduced basic research principles and types of study designs. B.B. explained the institutional review board approval process for the work group study, including issues of human subjects' protection. During this session, participants expressed concern about cancer research being fragmented, with many organizations competing for funds. Additionally, participants highlighted the need to share new cancer research findings in a comprehensive manner and stressed the need for improved communication about research with community members.

### Step 4: Describe the big picture: state cancer plans

Participants were unaware of state cancer plans. They suggested this might be because of the distance between mountain counties and state capitals, where, they perceived, health care decisions are made. In session 4, State Comprehensive Cancer Control Plan representatives from Nashville, Tennessee, and Richmond, Virginia, explained their state plans. Participants asked questions, clarified information, and expressed concern that they did not know who from their communities had contributed to the state plans or served as local members of statewide coalitions. They explained that a lack of involvement in statewide cancer coalitions might reflect the Appalachian sense of distrust toward government and the regional history of communities "taking care of our own" rather than relying on state help.

### Step 5: Use what is learned to promote community understanding and action

Work group members agreed to formulate their own cancer control messages and initiatives on the basis of what they learned from sessions 1 through 4. Session 5 provided participants an opportunity to design and lead local education activities in their communities. Each participant designed and delivered cancer control messages by using his or her preexisting social networks. Messages were disseminated in various ways: through their professions (eg, schools, libraries, funeral homes), their personal contacts (eg, civic groups, social groups, churches), and local community media (eg, newspaper editorials, articles). The most frequently communicated message was about high cancer mortality rates in Appalachia. Participants enthusiastically communicated messages through creative grassroots community approaches: talking about cancer disparities with a community quilting group, writing letters to their physicians to support hiring patient advocates, designing posters that target men at a local lawnmower repair shop, distributing pamphlets at employment offices, producing radio segments, and publishing letters to editors. Participants reported feeling like informed messengers, and stated that work group participation gave them something to take back to communities to potentially improve quality of life.

## Impact

The work groups themselves, and particularly the development of a successful process to convene them, have been beneficial to the communities. First, individual work group participants are now more engaged with ETSU researchers in cancer control activities, helping to educate state and federal representatives on the cancer experience in central Appalachia. Participants have continued to use information from the work groups to guide their personal actions in both professional and interpersonal communications. Second, new community awareness of the state cancer plans is evident; local coalitions are better linked with state coalitions. Third, work group participants have more faith in the research process and the positive outcomes that can arise from CBPR.

## Conclusion

The Appalachian Community Cancer Research Review Work Groups demonstrate how state cancer coalitions can help communities be involved in implementing state cancer plans locally. Busy community leaders will make time and use inventive ways to translate and communicate cancer information that concerns them to their communities. Bringing researchers and communities together through participatory processes yields rich interpretations that might otherwise escape recognition. Summaries of our interpretations and findings are also documented elsewhere (4,12).

The effort was not without challenges. Most community leaders wished to focus on solutions rather than understanding research, resulting in a recruitment challenge. Attendance at sessions was good, but individual illness and work demands prevented some participants from fully participating.

The work group approach represents a long-term investment in cancer control. Through short-term time commitments, participants now feel more empowered to use data, research findings, and health communication to help their communities. Although general awareness of state cancer plans and state coalitions may be limited at the local level, community leaders, such as the work group participants, are interested in the efforts and want to participate more. Connecting underserved communities that suffer from high cancer incidence and mortality rates with state and national programs will take persistence and small-scale investments like the Community Cancer Research Review Work Groups.

## Figures and Tables

**Table.** Appalachian Community Cancer Research Review Work Groups Session Breakdown, 2006

Session No.	**Session Intent**	**Participants' Role**
**1**	Report cancer disparities findings from RACDP and ARC studies.	Interpret research and generate questions from community perspective.
**2**	Describe communication issues in cancer care.	Identify health literacy and patient-provider communication influences on cancer outcomes.
**3**	Explain research methods and human subjects protection.	Define community sociocultural issues in cancer research.
**4**	Describe state cancer control plans and coalitions.	Discuss elements, process, and opportunities for community involvement.
**5**	Conduct work group member dissemination activity.	Present findings from within their own social networks.
**6**	Review summary of work groups.	Define what makes experiences with cancer in Appalachia unique.

Abbreviations: RACDP, Rural Appalachian Cancer Demonstration Program; ARC, Appalachian Regional Commission.
